# IL-8 predicts early mortality in patients with acute hypercapnic respiratory failure treated with noninvasive positive pressure ventilation

**DOI:** 10.1186/s12890-017-0377-7

**Published:** 2017-02-08

**Authors:** Brynja Jónsdóttir, Åsa Jaworowski, Carmen San Miguel, Olle Melander

**Affiliations:** 1 The Department of Clinical Sciences Malmo, Faculty of Medicine, Lund University, Lund, Sweden; 2 Department of Lung- and Allergy Medicine, Skåne University Hospital, Malmö, Sweden; 3 Department of Internal Medicine and Emergency Medicine, Skåne University Hospital, Malmö, Sweden

**Keywords:** Acute Respiratory Failure, Noninvasive Positive Pressure Ventilation, Short-time Mortality, Interleukin-8, Growth Differentiation Factor 15

## Abstract

**Background:**

Patients with Acute Hypercapnic Respiratory Failure (AHRF) who are unresponsive to appropriate medical treatment, are often treated with Noninvasive Positive Pressure Ventilation (NPPV). Clinical predictors of the outcome of this treatment are scarce. Therefore, we evaluated the role of the biomarkers IL-8 and GDF-15 in predicting 28-day mortality in patients with AHRF who receive treatment with NPPV.

**Methods:**

The study population were 46 patients treated with NPPV for AHRF. Clinical and background data was registered and blood samples taken for analysis of inflammatory biomarkers. IL-8 and GDF-15 were selected for analysis, and related to risk of 28-day mortality (primary endpoint) using Cox proportional hazard models adjusted for gender, age and various clinical parameters.

**Results:**

Of the 46 patients, there were 3 subgroup in regards to primary diagnosis: Acute Exacerbation of COPD (AECOPD, *n* = 34), Acute Heart Failure (AHF, *n* = 8) and Acute Exacerbation in Obesity Hypoventilation Syndrome (AEOHS, *n* = 4). There was significant difference in the basic characteristic of the subgroups, but not in the clinical parameters that were used in treatment decisions. 13 patients died within 28 days of admission (28%). The Hazard Ratio for 28-days mortality per 1-SD increment of IL-8 was 3.88 (95% CI 1.86–8.06, *p* < 0.001). When IL-8 values were divided into tertiles, the highest tertile had a significant association with 28 days mortality, HR 10.02 (95% CI 1.24–80.77, p for trend 0.03), compared with the lowest tertile. This correlation was maintained when the largest subgroup with AECOPD was analyzed. GDF-15 was correlated in the same way, but when put into the same model as IL-8, the significance disappeared.

**Conclusion:**

IL-8 is a target to explore further as a predictor of 28 days mortality, in patients with AHRF treated with NPPV.

## Background

As Chronic Obstructive Lung Disease continues to be a leading cause of mortality and morbidity worldwide, the importance of choosing the most appropriate treatment for each patient is vital [1, 2]. In Acute Hypercapnic Respiratory Failure (AHRF) due to Acute Exacerbation of COPD (AECOPD), Noninvasive Positive Pressure Ventilation (NPPV) has been shown to reduce the need for endotracheal intubation, the length of hospital stay, and the in-hospital mortality rate, in patients who are unresponsive to acute appropriate medical therapy [3–8]. Sixty-day survival benefit has even been shown in patients with acute-on-chronic respiratory failure [9]. Patient selection is important, and the treatment is most effective in the early stages of acidosis [10–13]. Factors that have been show to predict NPPV failure in patients with AECOPD (resulting in endotracheal intubation or death) are severe acidosis (pH <7.25), low Glasgow Coma Scale scores, high respiratory rate and high APACHE-II score [14]. Even lack of improvement within the first hour after initiation of NPPV is a negative prognostic factor in patients with various underlying causes of AHRF [15].

NPPV is even effective in some other causes of respiratory failure, such as acute heart failure, pneumonia in COPD patients and infections in immunocompromised patients [16–20]. Berg et al. stated in a review article, that in patients with acute heart failure treated with nonvasive ventilation, many studies show some degree of benefit in regards to relief of respiratory distress, lower intubation rates or decreased mortality. There is no difference between NPPV and CPAP (continuous positive airway pressure), the latter being the first treatment option in acute cardiogenic pulmonary edema, but NPPV is recommended if there is any evidence of hypercapnia or if the patient remains in distress despite treatment with CPAP [21] Both NPPV and CPAP are also commonly used in exacerbations of obesity hypoventilation syndrome [22]. A comparison of NPPV treatment in patients with AHRF in AECOPD or Obesity Hypoventilation Syndrome (OHS) showed similar effectiveness regarding survival, in-hospital mortality, and length of hospital stay [23]. There is a lack of randomized clinical trials for OHS patients with AHRF, but the treatment is considered safe and effective, even if acidosis prevails generally longer than in patients with AECOPD [24].

COPD is associated with low-grade systemic inflammation. The search for biomarkers to predict outcome in COPD patients, both during exacerbations and in stable condition, has been extensive in the last few years [25, 26]. To our knowledge, no study has been published that has evaluated the roll of inflammatory biomarkers to predict short term mortality in patients with AHRF (with various underlying causes) treated with NPPV.

In order to improve risk stratification of patients with acute dyspnea, we recently assessed inflammatory biomarkers and their predicting value for mortality in patients that came to the ER with acute dyspnea, and found that in this group, Interleukin-8 (IL-8) and Growth Differentiation Factor 15 (GDF-15) strongly and independently predict 90-days mortality, individually and as an aggregated score [27]. IL-8 is produced by various cells in the inflammatory pathway. Among other things, it induces the migration of neutrophils to the airway [28]. GDF-15 is a regulatory protein in the inflammatory pathway and is also produced by various cells in response to oxidative and inflammatory factors [29]. Based on the strong association between IL-8 and GDF-15 and mortality in patients with acute dyspnea in our previously published study [27], we hypothesize that these two inflammatory markers may add clinically meaningful information regarding 28-day mortality in AHRF patients receiving NPPV treatment.

## Methods

### Study population

During the period January 2014 to June 2014 we enrolled adult patients with acute respiratory failure that had clinical indication for treatment with NPPV according to local clinical guidelines, in the Intermediate Emergency Care Department at Skane University Hospital in Malmö, Sweden. The hospital serves a catchment area of approximately 400,000 inhabitants. We did not perform a power calculation as no prior suggested effect size for the association between IL-8 and GDF-15 on 28-day mortality in AHRF patients on NPPV treatment exist, on which the power calculation could be based. Written informed consent was obtained from all patients or their next of kin.

We included all patients that received treatment with NPPV for AHRF, decided by the attending physicians and regardless of the underlying disease. Patients with neurological disease or sepsis as the main cause of respiratory failure were excluded. Participation did not intervene with the treatment itself and the research personal was not responsible for the medical treatment in any way. Thus, the study was observational and prospective. As the clinical value of NPPV treatment in AHRF is nonmistakable according to various studies, it was not possible to have a control group deprived of the treatment [5, 8, 9, 16, 19, 23].

The study was approved by the Regional Ethics Board of Lund, Sweden and followed the precepts established by the Declaration of Helsinki.

### Clinical parameters and follow-up

Vital parameters were obtained before and during the treatment at several previously decided time points (0, 1, 4, and 12 h after the start of treatment and venous blood samples and arterial blood gases (ABG) were taken. Vital parameters recorded were body temperature, peripheral oxygen saturation (SpO_2_), heart rate, blood pressure, respiratory rate and degree of consciousness according to the “Reaction Level Scale” (RLS) [30]. The ABGs were analyzed immediately on a ABL800 Flex (Radiometer, Copenhagen, Denmark), while the venous blood samples were frozen and stored at −80 °C for later analysis of biomarkers, after having separated serum and plasma.

All the patients received Bilevel NPPV treatment with Trilogy100 and a suitable NPPV mask (Respironics, Murrysville, Pennsylvania/USA), using S/T mode. Expiratory positive airway pressure (EPAP) was set to 5 cm H_2_O. Inspiratory positive airway pressure (IPAP) was automatically regulated with average volume assured pressure support (AVAPS), so that a pressure between 10 and 25 cm H_2_O was applied to obtain a goal tidal volume of 8 ml/kg. Backup respiratory rate was set to 10 breaths/min. Oxygen was applied as needed with a goal SpO_2_ of 88–90%. The patients were monitored closely during the treatment. The treatment was only stopped for shorter periods, for example during meals. The physicians on call decided when the treatment was discontinued. Details about treatment length and installations was obtained from the Directview Program (Respironics, Murrysville, Pennsylvania/USA).

The patients consented to have their medical history and current medication obtained through the journal database of the hospital. Smoking habits, employment history and marital status was obtained through interview.

### Biomarker measurement

Based on our previous findings in risk stratification of patients with acute dyspnea [27], we selected to study IL-8 and GDF-15 which were measured in frozen plasma samples using the Proseek Multiplex CVD 1 biomarker panel (Olink Bioscience, Uppsala, Sweden). The method is a multiplex immunoassay based on a Proximity Extension Assay [31]. All assay characteristics including detection limits and measurements of assay performance and validations are available from the manufacturer’s webpage [32].

### Endpoint

The primary endpoint in the current study was defined as death within 28 days after admission to the ER. We confirmed deaths and date of death using the Swedish National civil registry.

### Statistical analysis

All statistical analysis was performed with IBM SPSS statistics version 21 (SPSS Inc., Chigago, IL, USA). In univariate analyses we used Kruskal-Wallis test to analyze continuous variables, and expressed data as medians and interquartile ranges. For categorical variables, Fishers exact test was used and data was expressed as numbers and percentages. We used Cox proportional hazards model to relate baseline variables to risk of death during 28 days of follow-up. Biomarkers levels were transformed with the natural logarithm and expressed as hazard ratios (95% confidence interval) on a standardized scale (per 1 standard deviation increment) as well as in tertiles, with the lowest tertile as the reference group. We adjusted for age and gender (model 1) and age, gender and C-reactive protein (CRP) (model 2) and also entered multiple potential confounders using backward stepwise Wald selection with a retention *P*-value >0.10 (model 3). Finally, crude Kaplan-Meier plots for tertiles of IL-8 levels were plotted. All tests were two-sided and a *p*-value of <0.05 was considered statistically significant.

## Results

### Patient characteristics

Fifty-one patients were enrolled during the study time, but five patients were excluded because of withdrawal of consent (*n* = 3), presence of neurological disease (*n* = 1) and sepsis (*n* = 1) as main underlying causes of AHRF, leaving forty-six patients in the analysis. No patient was intubated during the hospital stay. Chest radiographies were performed bedside on 39 patients (85%). 2 patients had radiological evidence of possible consolidation, both of which survived the follow up time of 28 days. There was no significant correlation between radiologial evidence of heart failure or consolidation, and 28 days mortality (data not shown). All patients were evaluated by attending physicians regarding vital status, and in 33 patients (72%), the “Do Not Resuscitate” order was made and recorded in the medical journal, according to local guidelines. Twenty-four patients (52%) were evaluated not to be eligible for ICU ward. Thirteen patients died within 28 days after admission (28%), of whom 10 patients died during the hospital stay. The median length of stay was 7 days (IQR 4–11 days).

Before analysis of data, the medical records were examined by an internist and a primary discharge diagnosis was made. There were 3 subgroups of patients in regards to primary diagnosis: Acute Exacerbation of COPD (AECOPD), Acute Heart Failure (AHF) and Acute Respiratory failure in OHS (AEOHS). To evaluate if there was difference between the groups, clinical characteristics were compared between the groups (Table 1). There were significant group differences in age, BMI and smoking status. While the difference between the subgroups lay within the basic characteristics of the group population, but not in regards of variables which determined NPPV treatment, the assumption was made that all results could be analyzed as one group of patients with AHRF. Analyses were nonetheless also made on the largest subgroup of patients with AECOPD, as described below.

**Table 1:** Characteristics of the patients, as a whole group and divided into subgroups

	Whole group	AECOPD	AHF	AEOHS	*P* value^b^
General characteristics					
Number of patients	46	34	8	4	
Age years: median (IQR)	77.1 (68.7–84.0)	76.9 (68.8–83.9)	82.3 (77.7–86.8)	65.4 (60.5–73.0)	*0.035*
BMI kg/m^2^: median (IQR)	23.4 (20.5–36.1)	24.0 (18.8–28.2)	27.7 (21.7–39.2)	46.6 (39.3–55.2)	*0.004*
Gender female %	65% (30/46)	65% (22/34)	63% (5/8)	75% (3/4)	0.054
Active or ex-smokers %	87% (40/46)	97% (33/34)	63% (5/8)	50% (2/4)	*<0.001*
FEV1%: median (IQR)	31 (24–43)	29 (22–36)	47^a^	43^a^	0.058
Variables related to AHRF					
pH: median (IQR)	7.28 (7.24–7.36)	7.31 (7.24–7.37)	7.24 (7.10–7.31)	7.30 (7.25–7.35)	0.17
pO_2_ kPa: median (IQR)	7.45 (6.33–8.73)	6.85 (6.10–8.48)	7.50 (5.60–9.58)	8.40 (8.23–10.68)	0.24
pCO_2_ kPa: median (IQR)	8.75 (7.78–10.5)	8.90 (7.78–10.35)	8.05 (6.38–10.73)	10.05 (8.45–11.73)	0.30
Respiratory rate bpm: median (IQR)	26 (20–29)	26 (20–29)	24 (22–29)	25 (21–27)	0.89
CRP mg/L: median (IQR)	15.5 (8.3–76.5)	36.5 (9.7–93.0)	8.7 (6.4–11.0)	12.5 (7.5–37.0)	0.076
Lactate mmol/L: median (IQR)	1.40 (0.80–2.75)	1.10 (0.80–2.10)	3.80 (2.78–5.63)	1.20 (0.80–2.35)	0.09
NPPV use first 4 h: median (IQR)	3.57 (3.50–4.00)	3.67 (3.50–4.00)	3.50 (2.50–4.00)	3.75 (3.50–4.00)	0.67

*IQR* interquartile range, *AECOPD* acute exacerbation of COPD, *AHF* acute heart failure, *AEOHS* acute exacerbation of obesity hypoventilation syndrome, *BMI* body mass index, *FEV1* forced expiratory volume in 1 s, *CRP* C-reactive protein, *NPPV* noninvasive positive pressure ventilation

^a^variable number too small to analyse IQR

^b^We used Kruskal-Wallis test for all but gender and smoking status, there we used Fisher´s exact test

### IL-8 and GDF-15

The biomarkers IL-8 and GDF-15 at admission before treatment was started, showed significant association with 28 days mortality through adjusted Cox proportional hazard models 1 (age and gender adjusted) and 2 (age, gender and CRP adjusted) (Tables 2 and 3). Each 1 SD increment of IL-8 was associated with almost fourfold increased risk of 28 days mortality, and for GDF-15 the increase in risk was almost threefold (Tables 2 and 3). Even CRP alone (adjusted for age and gender) was analyzed in model 2, and was an independent risk factor for 28 days mortality (HR 1.61 (1.08–2.41), p 0.02). In model 3, where additional parameters (BMI and blood analysis (pH, pO_2_, pCO_2_, lactate) as well as respiratory rate at start of treatment and primary discharge diagnosis) were entered in a backward stepwise elimination model (data missing on *n* = 13), both IL-8 (*p* = 0.015) and GDF-15 (*p* = 0.008) remained significantly related to risk of 28-day mortality. The independent significant association between IL-8 or GDF-15 and 28-day mortality remained after exclusion of the two patients with consolidation on chest radiograph (data not shown).

**Table 2:** Relationship between Interleukin-8 (IL-8) and risk of 28-day mortality

IL-8 on admission vs 28 days follow-up death (Model 1 and 2)						
	Continuous IL-8 analysis (per SD increment)	*P*-value	Tertile 1	Tertile 2	Tertile 3	P for trend
N/N events^b^	46/13		15/1	16/3	15/9	
HR (95% CI) (age and gender adjusted)	3.88 (1.86–8.06)	*<0.001*	1.0 (ref)	2.79 (0.29–26.89)	10.02 (1.24–80.77)	*0.009*
HR (95% CI) (age, gender and CRP adjusted)^a^	3.76 (2.02–7.03)	*<0.001*	1.0 (ref)	3.11 (0.32–29.93)	13.47 (1.70–106.91)	*0.003*

*SD* standard deviation, *HR* hazard ratio, *CI* confidence interval, *CRP* C-reactive protein

^a^Backward elimination model

^b^death within 28 days from admission

**Table 3:** Relationship between Growth Differentiation Factor 15(GDF-15) and risk of 28-day mortality

GDF-15 on admission vs 28 days follow-up death (Model 1 and 2)						
	Continuous GDF-15 analysis (per SD increment)	*P*-value	Tertile 1	Tertile 2	Tertile 3	P for trend
N/N events^b^	46/13		15/1	16/3	15/9	
HR (95% CI) (age and gender adjusted)	2.76 (1.37–5.56)	*0.004*	1.0 (ref)	1.21 (0.19–7.77)	3.48 (0.54–22.34)	0.124
HR (95% CI) (age, gender and CRP adjusted)^a^	3.48 (1.78–6.80)	*<0.001*	1.0 (ref)	1.14 (0.16–8.21)	2.65 (0.36–19.41)	*0.036*

*SD* standard deviation, *HR* hazard ratio, *CI* confidence interval, *CRP* C-reactive protein

^a^Backward elimination model

^b^death within 28 days from admission

**Fig. 1:**
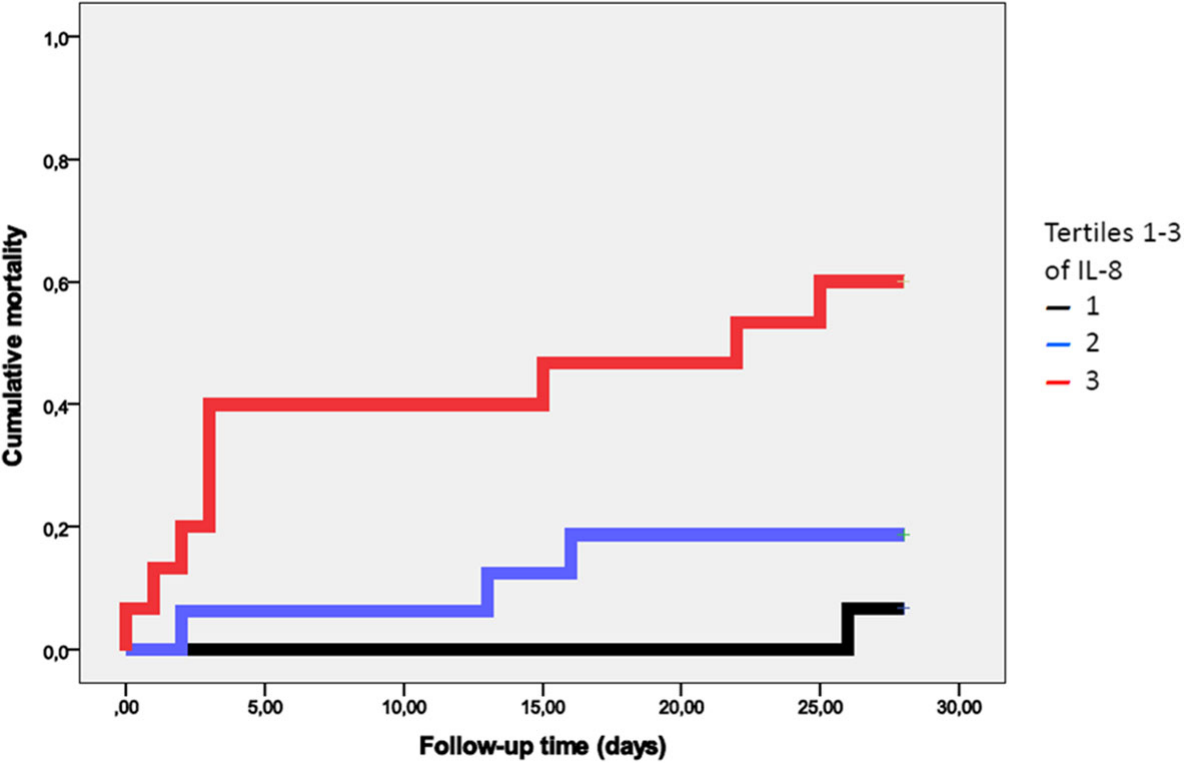
Kaplan-Meier plot showing cumulative mortality during 28 days of follow-up. Tertile 1 denotes the lowest values of IL-8; and Tertile 3 the highest values

IL-8 tertiles were entered into model 1 and 2 adjusted Cox proportional hazard model, patients in the highest tertile showed a significant 10-fold and 13-fold, respectively, increased risk of 28 days mortality, as compared to the lowest tertile (Table 2). The event rate in IL-8 tertiles according to a Kaplan-Meier plot is shown in Fig. 1. When GDF-15 tertiles were analyzed in the same manner, there was no significant association in model 1, but in model 2 there was a significant association with a 2,5 fold increased risk for mortality for the highest tertile as compared to the lowest (Table 3).

To evaluate if both IL-8 and GDF-15 were independent risk factors, we simultaneously entered them as continuous variables into model 1 and found that only IL-8 remained significantly associated, with HR 3.38 (95% CI 1.35–8.43, *p* = 0.009) per 1 SD increment.

Being the largest group, we then analyzed the group of patients with AECOPD as their primary diagnosis (*n* = 34) separately. Both IL-8 and GDF-15 at admission showed significant association with 28 days mortality in models 1–2 with approximately fourfold and threefold increased risk per 1 SD increment (Table 4). When simultaneously entered into model 1, only IL-8 remained significantly associated with HR 4.31 (95% CI 1.57–11.84, *p* = 0.005).

**Table 4:** Relationship between IL-8/GDF-15 and risk of 28-day mortality in a subgroup of patients with AECOPD

IL-8 and GDF-15 on admission vs 28 days follow-up death – AECOPD subgroup				
	IL-8		GDF-15	
	Continuous analysis (per SD increment)	*P*-value	Continuous analysis (per SD increment)	*P*-value
N/N events^b^	34/11		34/11	
HR (95% CI) (age and gender adjusted)	4.16 (1.84–9.39)	*0.001*	3.10 (1.30–7.40)	*0.011*
HR (95% CI) (age, gender and CRP adjusted)^a^	4.34 (2.05–9.19)	*<0.001*	3.28 (1.56–6.88)	*0.002*

*AECOPD* acute exacerbation of COPD, *SD* standard deviation, *HR* hazard ratio, *CI* confidence interval, *CRP* C-reactive protein

^a^Backward elimination model

^b^death within 28 days from admission

## Discussion

Our findings suggest that IL-8 is a target to explore further as a biomarker in predicting 28 days mortality, in patients with AHRF (Acute Hypercapnic Respiratory Failure) and AECOPD (Acute Exacerbation of COPD) who receive treatment with NPPV (Noninvasive Positive Pressure Ventilation). The results were driven by COPD patients, so further studies are needed not only to replicate our findings but also to test if the our findings are valid in the comparably small subgroup of non-COPD patients.

NPPV is a well-known treatment option for patients with AHRF. Earlier studies have shown that in patients with AECOPD, NPPV can reduce the risk of intubation, reduce in-hospital mortality and shorten the hospital stay [3–7]. Its use has been growing and more data has appeared supporting the positive effects of the treatment, even in other types of respiratory failure [21]. The treatment is costly in terms of technical equipment and surveillance, and not all patients can tolerate the treatment or benefit from it [17]. Even if there are clinical tools that have been shown to help in predicting the outcome of NPPV treatment, the search for parameters that can provide additional help in treatment decisions is important, both in regard to costs and patient comfort [12–14, 33].

Because of its predictive value in relation to short term mortality, our findings imply that the inflammatory biomarker IL-8 could potentially be used in the initial evaluation of the patient with AHRF, to help choose the most appropriate treatment. This potential clinical use can be used on patients with AECOPD and even maybe in other types of AHRF (Acute Heart Failure or Acute Exacerbation of OHS). This is clinically important, as the main cause for the AHRF is not always clear when the patient arrives to the ER. GDF-15 showed similar results as IL-8, but our analysis implies that it is not an independent factor but dependent of IL-8 in this clinical setting. CRP did not influence the correlation between IL-8 and 28 days mortality, and it was even not effected by the confounding factors age, gender, BMI, blood analysis (pH, pO_2_, pCO_2_, lactate) as well as respiratory rate at start of treatment or primary discharge diagnosis (Table 2). A high value of IL-8 might thus prompt the physician to choose more invasive treatment such as intubation. As many forms of acute and chronic illnesses are characterized by enhanced inflammation, it is likely that high baseline values of IL-8 and GDF-15 reflects not only a more severe acute condition but most likely also more severe chronic illness.

IL-8 is a chemokine that induces the migration of neutrophils to the airway and affects degranulation [28]. Both sputum and serum IL-8 have been targets for research in regards to COPD mortality and exacerbations. A review by Koutsokera et al. summarized that IL-8 levels in spontaneous sputum in patients with AECOPD can be predictive of clinical severity, symptomatic recovery and even presence of bacterial infection and its eradication [34]. Agusti et al. identified 6 inflammatory biomarkers (including IL-8) that were related to systemic inflammation, and patients with COPD in which those biomarkers were elevated in serum showed increased all-cause mortality and exacerbation frequency, during three-year follow up time [35]. Lastly, Shafiak et al. showed that in patients with AECOPD, serum IL-8 had higher levels in non-responders of NPPV vs. responders, where failure was defined as termination of NPPV trial and initiation of invasive mechanical ventilation. IL-8 was however not correlated to presence of bacterial infection [36]. To our knowledge, that is the only prior study that has studied IL-8 levels and its correlation to AECOPD and NPPV treatment. The combination of these results support that IL-8 can possibly be used as a clinical predictor of response to NPPV treatment in patients with AECOPD, although to our knowledge, no prior study has focused on correlation between IL-8 in serum and short-term mortality in this patient group.

The GDF-15 protein is also a member of the inflammatory pathway, and is produced by multiple cells in response to oxidative and inflammatory factors. In recent years, studies have shown that GDF-15 levels can help in predicting mortality and adverse events in patients with cardiovascular diseases and other diseases [29]. Recent studies have even evaluated the connection between GDF-15 levels and AECOPD, in which patients with AECOPD had higher baseline blood levels of GDF-15 and it is regarded as an independent predictor of the presence of AECOPD [37, 38]. Our data did not confirm that serum GDF-15 was an independent prognostic factor for short-time mortality in patients with AHRF who receive NPPV treatment, but suggested that it is dependent of IL-8 levels. To our knowledge no other study has addressed the role of GDF-15 in this clinical setting.

The predictive value of IL-8 on 28 days mortality was independent of the traditional biomarker CRP and it had a comparatively stronger association, although CRP levels also had an independent correlation to the end-point. Our study does not allow any conclusions as to whether or not the relationship between IL-8 and short term mortality is causal, but in our opinion encourages further mechanistic studies to better understand this issue.

Our study has several limitations, the main one being the relatively small sample size, which also results in wide confidence intervals. Further studies are needed, which should include a larger group of patients with a larger spectrum of diagnoses and preferably even patients that are intubated if NPPV treatment is not successful. Another limitation is that only 85% of the patients did a chest radiography during the initial hours of the NPPV treatment. Nonetheless, the radiological results did not have any correlation to 28 days mortality.

The group of patients in this study had a high percentage of the “Do Not Resuscitate” order (72%), compared with an audit made by Roberts and al in UK (40%) [39]. A possible reason for this difference is that in the local guidelines, the admitting physician is obligated to make a resuscitation judgement if the patient is to be treated with NPPV. 28 days mortality was (28%) and can be regarded as more severely ill than groups of patients in some other studies in the field [8, 19], but similar to that reported in the UK audit (25% inhospital mortality) [39]. In this setting it is a challenge to recruit patients for research projects. Nonetheless, it is our opinion that more research in severely ill patients is necessary, to help clinicians make the choice between the use of demanding treatment such as NPPV or go directly to intubation. Our study did not include any patients that were later intubated so we cannot make any assumption as to how IL-8 values are effected by that. In our opinion it would be interesting to raise that question in further studies, and also evaluate the hypothesis that if IL-8 values do not become lower during noninvasive or invasive respiratory support, the prognosis would be poor in regards to short term mortality.

Another limitation of the study is the heterogeneity of the group. Subgroup analysis did not seem to influence the results though and the largest subgroup of patients, with AECOPD, have an even stronger association with the endpoint. Since the other two subgroups with AHF and AEOHS were so small in numbers, one should be careful to interpret the results in that clinical setting. The diagnosis of COPD and heart failure prior to admission came from the patients’ medical records, but some data regarding spirometry and echocardiography was not present. Nonetheless, the primary discharge diagnosis was assessed by the same internist in all cases, and the probability of wrong diagnosis seems minimal because of the large amount of clinical data available during the admission. In our opinion, the heterogeneity of the group reflects the clinical setting in the ER where the underlying medical condition resulting in respiratory failure is commonly unknown, and the results can be applied in that context. Further studies of patients with AHRF in regards of clinically important prognostic factors are relevant because the treatment with NPPV can be costly and difficult for patients, and existing patient selection tools do not always help in treatment decision.

## Conclusion

Our results show that IL-8 in serum is a target to explore further as a predictor of short-time mortality in patients with Acute Hypercapnic Respiratory Failure and Acute Exacerbation of COPD treated with Noninvasive Positive Pressure Ventilation. Its use in the initial assessment of this patient group should be addressed in studies with a larger number of patients but our result suggests a use of IL-8 as a tool to help physicians in treatment decisions.

## Abbreviations

ABG: arterial blood gas
AECOPD: acute exacerbation of chronic obstructive pulmonary disease
AEOHS: acute exacerbation of obesity hypoventilation syndrome
AHF: acute heart failure
AHRF: acute hypercapnic respiratory failure
APACHE-II: acute physiology and chronic health evaluation ii
AVAPS: average volume assured pressure support
BMI: body mass index
CI: confidence interval
COPD: chronic obstructive pulmonary disease
CPAP: continuous positive airway pressure
CRP: C reactive protein
EPAP: expiratory positive airway pressure
ER: emergency room
FEV1: forced expiratory volume in 1 second
GDF-15: growth differentiation factor 15
HR: hazard ratio
ICU: intensive care unit
IL-8: interleukin-8
IPAP: inspiratory positive airway pressure
IQR: interquartile range
NPPV: noninvasive positive pressure ventilation
OHS: obesity hypoventilation syndrome
RLS: reaction level scale
SD: standard deviation
